# Post-Mastectomy Radiotherapy for Breast Cancer Patients with T1-T2 and 1-3 Positive Lymph Nodes: a Meta-Analysis

**DOI:** 10.1371/journal.pone.0081765

**Published:** 2013-12-03

**Authors:** Yaming Li, Meena S. Moran, Qiang Huo, Qifeng Yang, Bruce G. Haffty

**Affiliations:** 1 Department of Breast Surgery, Qilu Hospital, Shandong University, School of Medicine, Ji'nan, Shandong, P. R. China; 2 Department of Therapeutic Radiology, Yale University School of Medicine, New Haven, Connecticut, United States of America; 3 Department of Radiation Oncology, University of Medicine and Dentistry of New Jersey, Robert Wood Johnson School of Medicine, and the Cancer Institute of New Jersey, New Brunswick, New Jersey, United States of America; University Medical Centre Utrecht, Netherlands

## Abstract

**Background:**

The role of post-mastectomy radiotherapy (PMRT) in patients with T1-2 and 1-3 positive lymph nodes remains controversial. The aim of this study is to investigate the possible benefits of PMRT for this subgroup.

**Methods:**

Three electronic databases were systematically quarried (Cochrane Library, MEDLINE, and EMBASE) for published studies evaluating the effects of PMRT on breast cancer patients with T1-T2 tumors with 1-3 positive lymph nodes. Of the 334 studies identified, information was available for 3432 patients from 10 clinical studies. Pooled relative risk estimates (RR) and overall survival (OS) were calculated using the inverse variance weighted approach, publication bias and chi-square test were also calculated.

**Results:**

From the 10 studies, the pooled RR (RRs) for locoregional recurrence (LRR) with PMRT was 0.348 (95% CI = 0.254 to 0.477), suggesting a significant benefit for PMRT to decrease the risk of LRR in patients with T1-T2 tumors and 1-3 positive nodes (p<0.05). Reporting bias ( Begg’s p = 0.152; Egger’s p = 0.107) or significant heterogeneity (Cochran’s p = 0.380; I^2^ = 6.7%) were not detected. For further subset analysis, the RR for T1, N1-3+ tumors was 0.330 (95% CI = 0.171 to 0.639); for T2, N1-3+ tumors the RR was 0.226 (95% CI = 0.121 to 0.424). The pooled RR for overall survival (OS) was not significantly different between PMRT and no-PMRT group (1.051, 95% CI =1.001 to 1.104).

**Conclusions:**

Our pooled analysis revealed that PMRT significantly reduces the risk of LRR in patients with TI-T2 tumors with 1-3 positive nodes, and the magnitude of the LRR risk reduction is slightly greater for larger tumors. Our results suggest that PMRT should be considered for patients with T1/T2 tumors with 1-3 positive nodes to decrease the relatively high risk of LRR.

## Introduction

Post-mastectomy radiotherapy (PMRT) can reduce the risk for local-regional recurrence(LRR) and improve survival in breast cancer patients with positive nodes [1,2]. Randomized data from the DBCG 82 b & c trials have demonstrated that the addition of PMRT for node positive patients improves the 15-year overall survival by approximately 10% (p=0.015). Furthermore, in these trials, PMRT reduced the 15-year LRR rate from 27% to 4% (p<0.001) in patients with 1-3 positive nodes [1]. The role of PMRT for ‘high-risk’ breast-cancer patient, traditionally defined as tumor size >5cm, positive nodes≧4 or positive margins, in decreasing LRR has been well documented [2-4]. Overgaard et al conducted a randomized trial of radiotherapy after mastectomy in 1708 high-risk premenopausal women, which shows the probability of disease-free survival at 10 years was 48% among the women assigned to radiotherapy plus CMF and 34% among those treated only with CMF (P<0.001), with a similar magnitude of benefit in overall survival at 10 years from 45% without PMRT to 54 % with PMRT (P<0.001) [3]. However, in subgroup with T1-T2 tumors and 1-3 positive axillary lymph nodes (T1/T2, N1-3+), the use of PMRT still remains controversial [2,5-7]. In the 2001 American Society of Clinical Oncology (ASCO) PMRT practice guideline, the panel concluded that there was insufficient evidence to make recommendations regarding T1/T2,N1-3+ patients [7]. Furthermore, the St. Gallen consensus conference recommended PMRT only for women with a LRR risk of 20% or greater [8]. The ongoing randomized control trial SUPREMO (Selective Use of Postoperative Radiotherapy after Mastectomy) was designed to evaluate the results of chest wall irradiation in management of the patients underwent MRM with pT1-pT2 disease, and it may give us further guidance on the use of PMRT to this subgroup patients[9].

Though it has been demonstrated that LRR is an important endpoint for breast cancer that can ultimately impact disease-free and overall survival, the identification of prognostic factors for LRR have not been consistent. Several studies have attempted to identify subsets of patients within the T1/T2, N1-3+ cohort who may have a higher risk of LRR [10-12]. For example, Truong et al reported that age <45 years, >25% nodal ratio, a medial tumor location, and ER-negative status as factors independently associated with greater LRR risk [10]. Yang et al suggested that within this T1/T2, N1-3+ subset, negative ER status and presence of lymphovascular invasion conferred a higher risk of LRR [12]. Thus, further elucidation of this cohort for overall risk of LRR and identification of subgroups of patients at increased risk for LRR are warranted.

While several meta-analyses have been conducted to investigate the effects of PMRT on patient outcomes, none have specifically focused on T1/T2 N1-3+ patients [13-16]. Thus, the objective of this systematic review was to investigate the effects of PMRT on LRR and OS in this specific group of patients.

## Materials and Methods

### Search strategy

Three electronic databases (Medline, Embase and Cochrane library) were quarried with the inclusion dates of January 2000 to April 2013 to search the following terms: ’breast cancer[mesh]’, ‘radiotherapy[mesh]’, ’ lymph node[mesh]’, and ‘1-3’ or ‘one to three’ or ‘no more than 3’. Copies of all eligible studies were obtained and read. Each bibliography was also carefully examined to identify other eligible studies. If there was an overlap in the patient cohorts across more than one study, only data from the largest published report was utilized. Only studies published in English were included in this meta-analysis.

### Inclusion and Exclusion Criteria

Studies included in this meta-analysis had to meet all of the following criteria: (a) patients received mastectomy (with or without radiotherapy) were collected, (b) tumor characteristics had to be microscopic tumor size≦5cm, with 1-3 tumor positive nodes (pT1/T2pN1), (c) no previous neoadjuvant systemic therapy or radiation, (d) either retrospective or prospective data, (e) the article had to be published in English. The major exclusion criteria were as follows: (a) patients received breast-conserving therapy are not involved in our study, (b) no sufficient data within the article to allow for the estimation of a risk ratio (RR) with 95% confidence intervals (95% CI), (c) 5-year incidence of LRR cannot be collected, (d) involve patients who received previous neoadjuvant systemic therapy or radiation, (e) overlapping or republished studies.

### Data abstraction

Based on the inclusion criteria above, the following data parameters were extracted for each study: the name of the first author, year of publication, country of origin for the study, total number of patients analyzed, patient and tumor characteristics, ratio of adjuvant systemic therapy and radiation fractionation scheme. Information was carefully and independently extracted from all eligible publications by two of the authors, any disagreement between the researchers was resolved by discussions until a consensus was reached. If they failed to reach a consensus, a third investigator (an experienced professional breast surgeon) was consulted to resolve the dispute.

### Statistical Analysis

Stata V.11.0 software was utilized for all statistical analysis. The outcomes, RR, 95% confidence intervals (CIs) were all calculated, and associations of PMRT to patient outcomes was assessed. Pooled RRs were performed, using the Z-test to determine its statistical significance. Statistical heterogeneity was calculated by chi-square test and used a fixed-effect-model for I^2^ < 50%, and a random-effect-model for I^2^≧50%. Publication bias was calculated using the Begg test. For all tests, a probability level lower than 0.05 was considered statistically significant. All statistical tests were two-sided.

## Results

### Search Results

**Figure 1:**
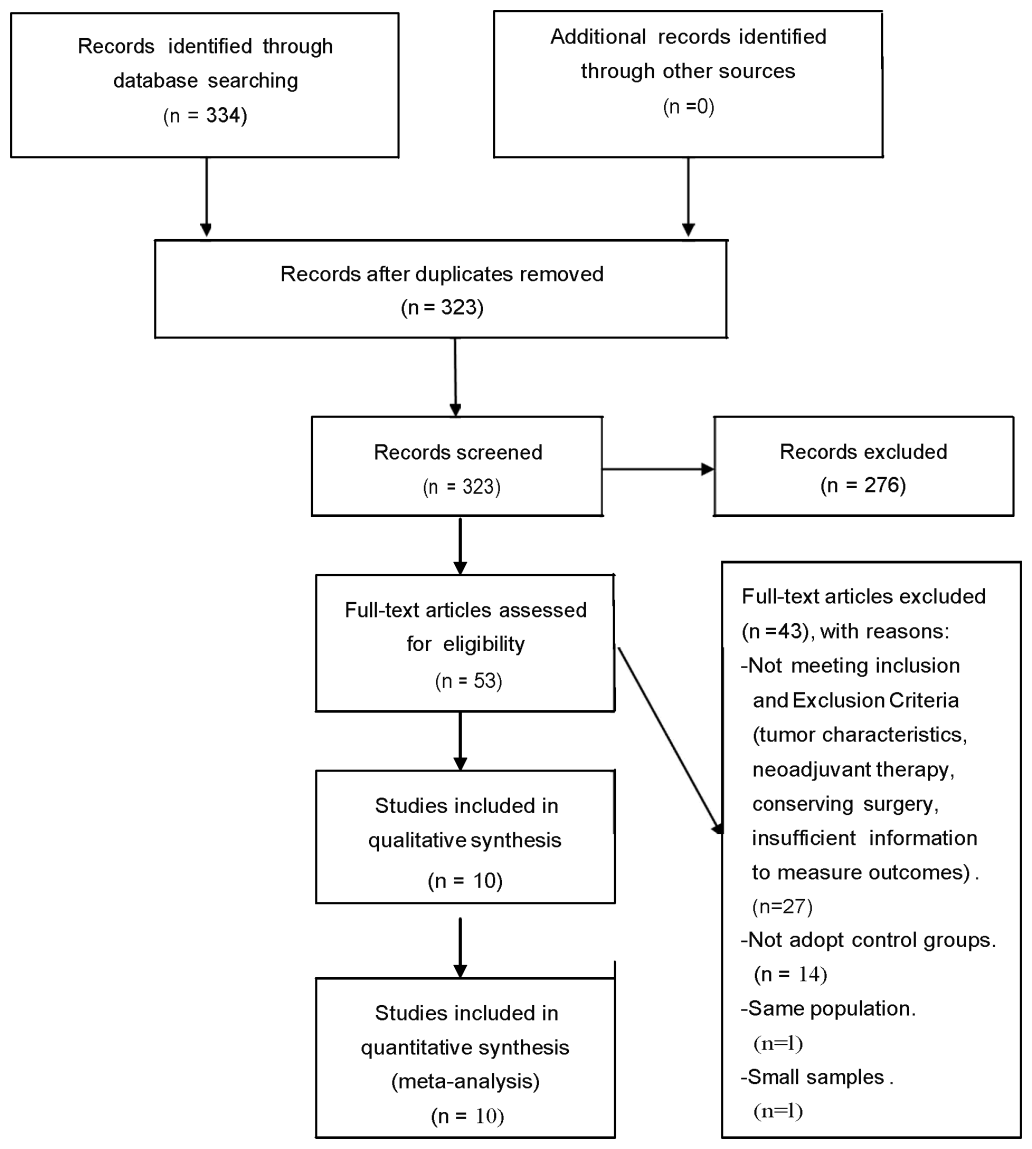
Flow diagram of this meta-analysis.

A total of 334 studies were initially identified based on the search criteria, only 11 studies met all inclusion/exclusion criteria, and 1 of the 11 studies was excluded because of the small sample size of patients who received PMRT (n= 12 patients). Ultimately, 10 studies met the inclusions criteria for this meta-analysis (Figure 1) [11,12,17-24].

Table 1 summarizes the characteristics of the trials included in this review. From the 10 studies, a total of 3432 patients were pooled, with 1478 (43.1%) having received PMRT and 1,954 (56.9%) who did not receive PMRT. By definition, all trials included patients treated with systemic therapy. All patients have performed axillary lymph node dissection (ALND) according to the articles, and radiation fractionation schemes were list in Table 2. The absolute LRR rate was 3.8% (n=56) in the PMRT cohort and 10.7% (210) in the cohort who did not receive PMRT.

**Table 1:** Clinical trials studying patients with T1-T2 and 1-3 positive nodes with or without PMRT.

**Study**	**Years of study**	**Country**	**No.of patients**	**Median age(y)**	**Median lymph nodes**	**Ratio ofT1 patients**	**Ratio of LRR**		**Ratio of adjuvant systemic therapy**	
							**PMRT**	**no-PMRT**	Chemotherapy	Hormonal therapy
Cosar, R 2011[17]	1999-2006	TURKEY	90	51	11	22/90	2/66	4/24	89/90	66/90
Song, Y. Z 2011[18]	2001-2006	CHINA	434	48	14	84/434	5/196	15/238	--------	--------
Duraker, N 2012[19]	1993-2002	TURKEY	575	48	14	185/575	18/452	15/123	511/575	416/575
Wu, S. G 2010[11]	1998-2007	CHINA	488	47	14	133/488	1/76	42/412	481/488	363/488
Yang, P. S 2010[12]	1991-2005	TAIPEI	544	48	--------	237/544	3/161	37/383	430/544	392/544
Zhang, Y. J 2009[20]	1998-2002	CHINA	217	45	13	---------	7/51	26/166	202/217	166/217
Chen, X 2013[21]	2000-2007	CHINA	101		--------	---------	2/18	21/83	--------	0/101
MacDonald,S.M 2009[22]	1990-2004	USA	238	54	14	123/238	0/73	10/165	168/238	186/238
Fodor, J 2003[23]	1983-1987	HUNGRAY	249	54	11	135/249	9/175	13/74	41/249	71/249
Wang, S. Y 2011[24]	2000-2003	CHINA	496	48	15	120/496	9/210	27/286	--------	278/496

PMRT= Post-mastectomy radiotherapy. LRR= Locoregional recurrence

**Table 2:** The radiation fractionation schemes in the 10 trails.

Study	Sites irradiated	Radiation Regimen		
		Dose (Gy)	Fraction	Time (weeks)
Cosar, R 2011[17]	CW, SC, AX	50	25	5
Song, Y. Z 2011[18]	CW+SC	46~50	23~25	4.6~5
Duraker, N 2012[19]	CW, SC, AX	50	25	5
Wu, S. G 2010[11]	CW, SC, AX, IMN	46~50	----------	----------
Yang, P. S 2010[12]	CW, SC, IMN	46~50	----------	----------
Zhang, Y. J 2009[20]	CW, SC, IMN	46~50	23~25	4.6~5
Chen, X 2013[20]	CW, SC, IMN	46~50	23~28	----------
MacDonald,S.M 2009[22]	CW, SC	48.3~55.9	----------	----------
Fodor, J 2003[23]	CW, AX, IMN,SC	42~52	----------	5
Wang, S. Y 2011[24]	CW, IMN, SC,	48~54	25	2.9~7.1

CW= chest wall; SC= supraclavicular lymph nodes; AX= axillary lymph nodes; IMN= internal mammary nodes

### Meta-analysis of PMRT Use and LRR

**Figure 2:**
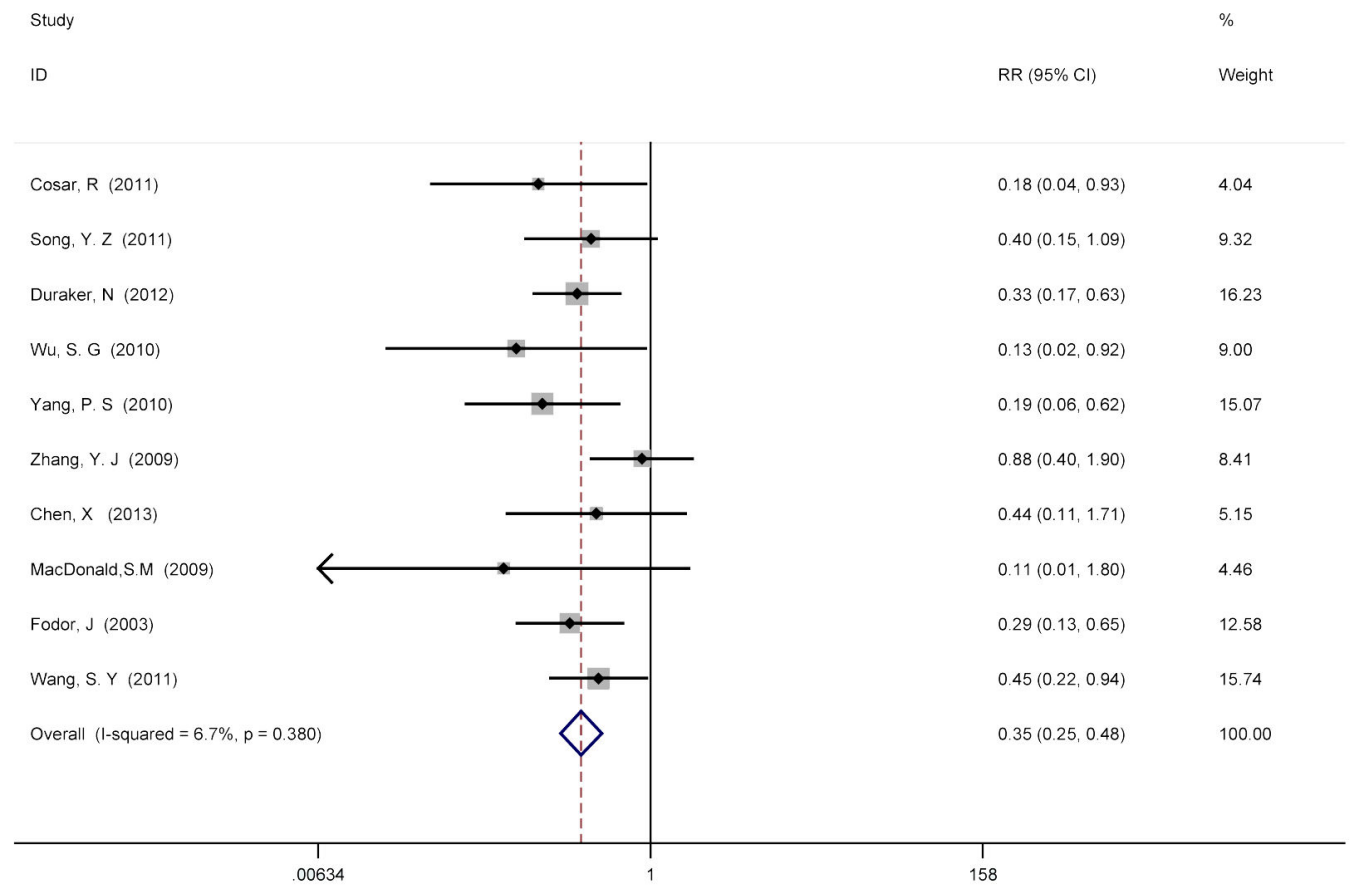
No PMRT versus PMRT after mastectomy surgery for T1/T2, N1-3+ patients on LRR. To investigate the role of PMRT in T1/T2, N1-3+ patients, LRR incidence was initially calculated as a function of PMRT utilization. The overall pooled RR of LRR risk in patients with PMRT versus no-PMRT from the 10 trails was 0.348 (95% CI = 0.254 to 0.477). Significant reporting bias (Begg’s p = 0.152; Egger’s p = 0.107) or heterogeneity between studies (Cochran’s p = 0.380; I^2^ = 6.7%) were not detected in these 10 studies[11,12,17-24].

To investigate the role of PMRT in T1/T2, N1-3+ patients, LRR incidence was initially calculated as a function of PMRT utilization. The overall pooled RR of LRR risk in patients with PMRT versus no-PMRT from the 10 trails was 0.348 (95% CI = 0.254 to 0.477), suggesting a statistical significant decrease in LRR risk in T1/T2, N1-3+ patients treated with PMRT (p<0.05) (Figure 2). Significant reporting bias (Begg’s p = 0.152; Egger’s p = 0.107) or heterogeneity between studies (Cochran’s p = 0.380; I^2^ = 6.7%) were not detected in these 10 studies[11,12,17-24].

### Meta-analysis of PMRT Use and LRR in T1 versus T2 patient subgroups

**Figure 3:**
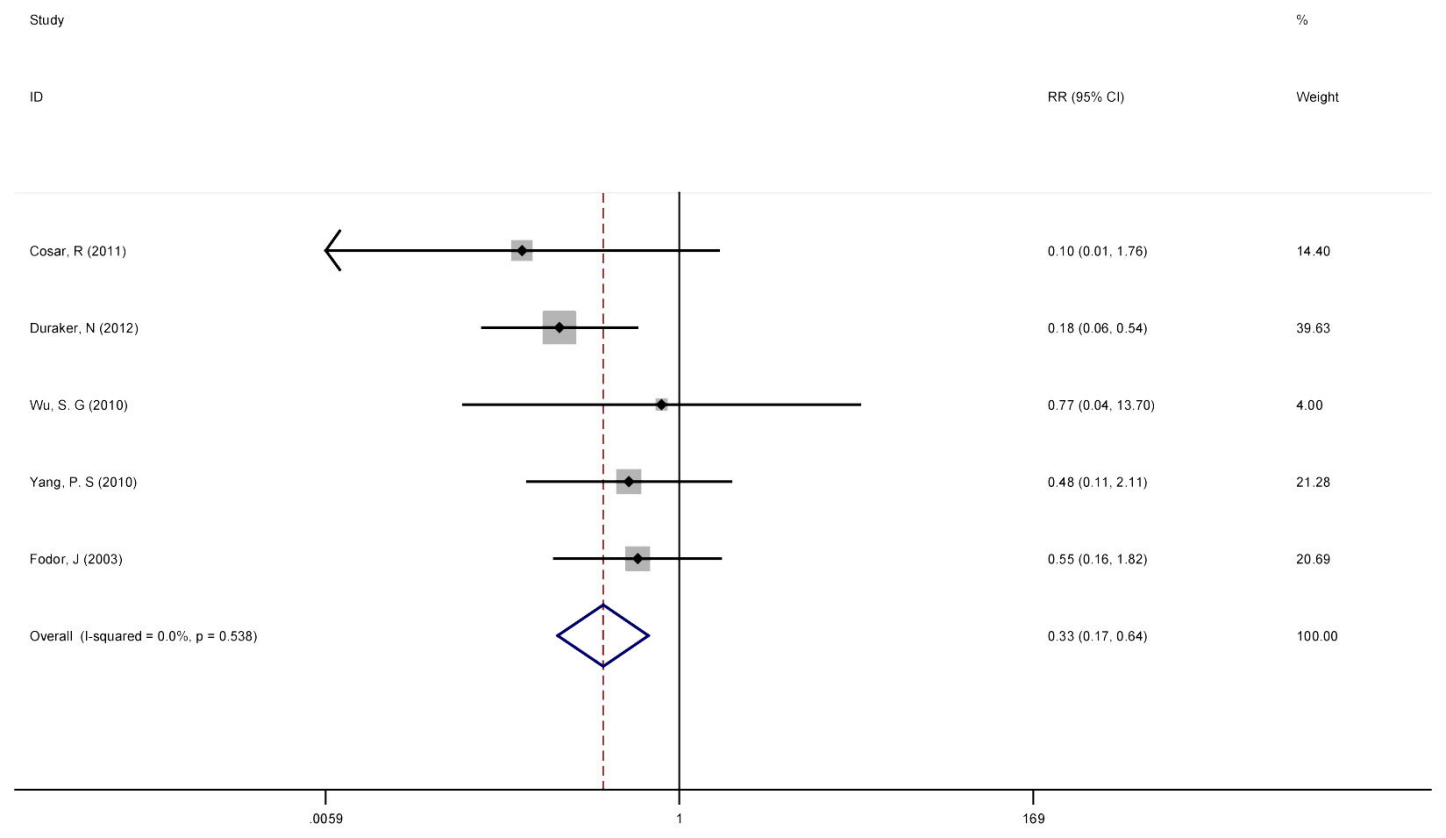
No PMRT versus PMRT after mastectomy surgery for the T1/N1-3+ subgroup on LRR. Five studies with detailed information were involved to investigate the role of PMRT in T1/N1-3+ patients. The pooled RR for LRR in patients with PMRT was 0.330 (95% CI = 0.171 to 0.639), and significant heterogeneity (Cochran’s p = 0.539; I^2^ = 0.00%) was not detected between the 5 studies included in this portion of the meta-analysis [11,12,17,19,23].

A subgroup analysis focusing on the incidence of LRR and the use of PMRT as a function of primary tumor size was conducted. Five studies had detailed information to be including in this portion of the analysis [11,12,17,19,23]. Patients were divided into two subgroups by tumor size: T1/N1-3+ and T2/N1-3+. For the T1/N1-3+ cohort, the pooled RR for LRR in patients with PMRT was 0.330 (95% CI = 0.171 to 0.639), suggesting a similar magnitude of benefit with the addition of radiation as was demonstrated from the larger cohort which included T1 and T2 patients from all 10 studies (Figure 3).Additionally, significant heterogeneity (Cochran’s p = 0.539; I^2^ = 0.00%) was not detected between the 5 studies included in this portion of the meta-analysis.

**Figure 4:**
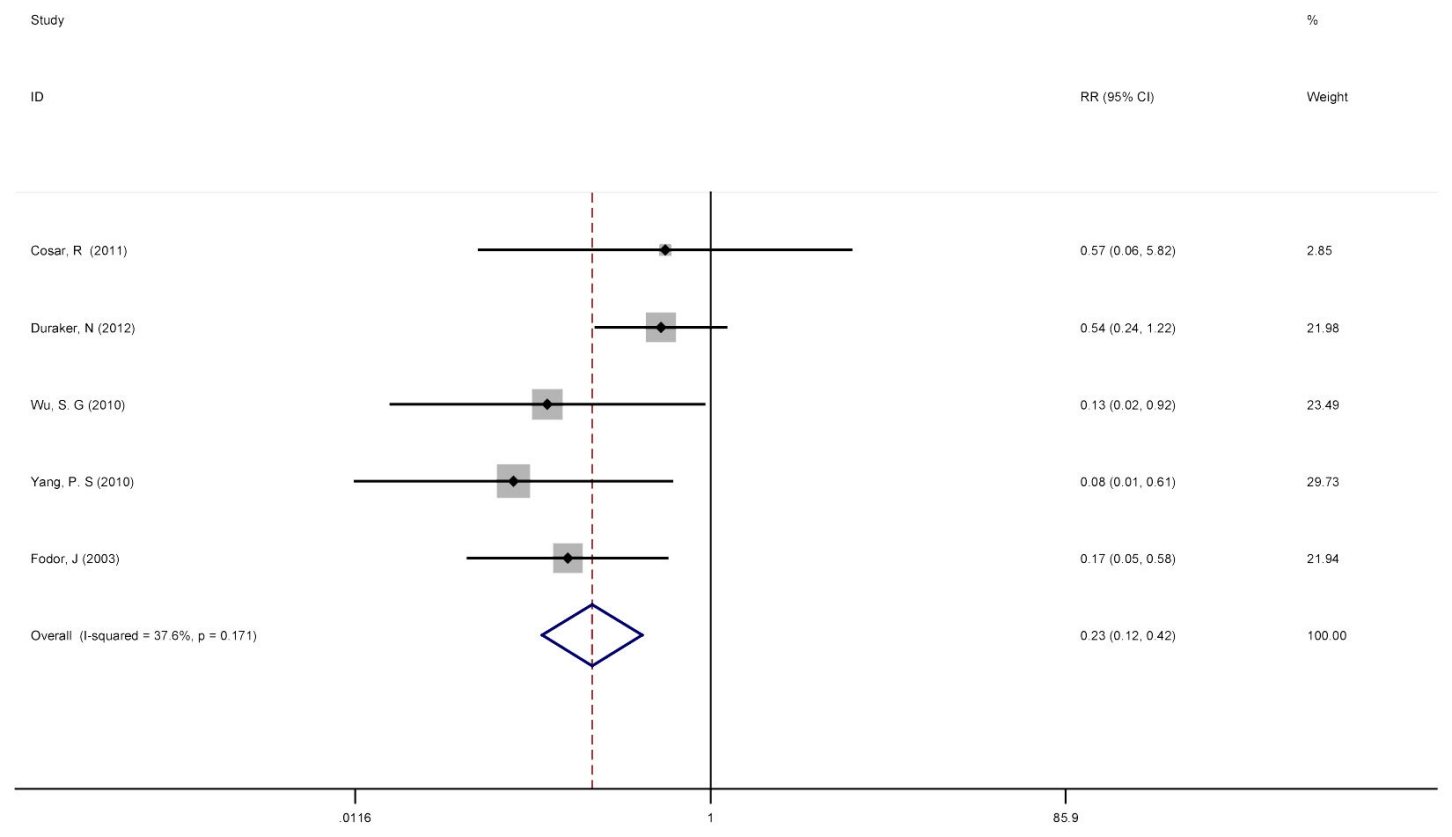
No PMRT versus PMRT after mastectomy surgery for the T2, N1-3+ subgroup on LRR. Five studies with detailed information were involved to investigate the role of PMRT in T2/N1-3+ patients. The pooled RR for LRR in patients with PMRT was 0.226 (95% CI = 0.121 to 0.424), and significant heterogeneity (Cochran’s p = 0.171; I^2^ = 37.6%) was not detected between the 5 studies included in this portion of the meta-analysis [11,12,17,19,23].

For T2/N1-3+ patients, the pooled RR for LRR with PMRT from these same 5 trials was 0.226 (95% CI = 0.121 to 0.424), demonstrating a slightly larger benefit for T2 tumors with the use of PMRT than for T1/N1-3+ patients or the T1/T2, N1-3+ combined cohort (Figure 4). Again, significant heterogeneity (Cochran’s p = 0.171; I^2^ = 37.6%) was not detected in this portion of the meta-analysis.

### Meta-analysis of PMRT Use and OS

**Figure 5:**
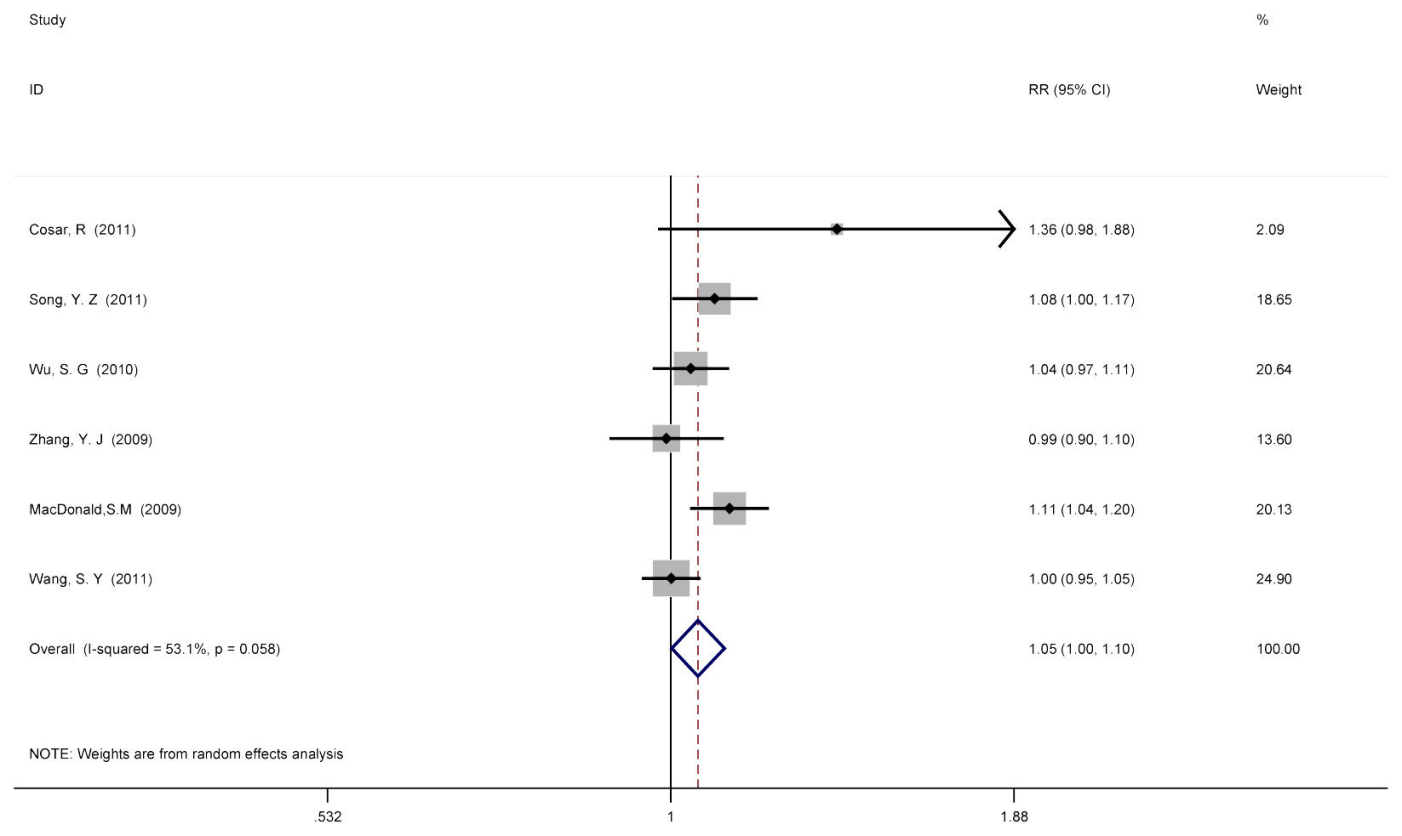
No PMRT versus PMRT after mastectomy surgery for T1/T2, N1-3+ patients on OS. To investigate the role of PMRT in T1/T2, N1-3+ patients on OS, analysis was conducted using available information from 6 of the 10 studies[11,17,18,20,22,24]. Random-effect model was chosen to estimate the RRs after pooled in view of the detected heterogeneity among these studies (Cochran’s p = 0.058; I^2^ = 53.1%), and the overall pooled RR of OS in patients with PMRT versus no-PMRT from the 6 trails was 1.051 (95% CI =1.001 to 1.104).

To investigate the role of PMRT in T1/T2, N1-3+ patients on OS, analysis was conducted using available information from 6 of the 10 studies[11,17,18,20,22,24]. Table 3 summarized the information of the 6 trials included in this analysis. At last, the overall pooled RR of OS in patients with PMRT versus no-PMRT from the 6 trails was 1.051 (95% CI =1.001 to 1.104) (Figure 5), and random-effect-model was used for the detected heterogeneity between studies (Cochran’s p = 0.058; I^2^ = 53.1%). 

**Table 3:** The presence of OS in the 6 studies.

**Study**	**Ratio of OS**	
	**PMRT**	**no-PMRT**
Cosar, R 2011[17]	10/66	9/24
Song, Y. Z 2011[18]	21/196	42/238
Wu, S. G 2010[11]	5/76	41/412
Zhang, Y. J 2009[20]	5/51	15/165
MacDonald,S.M 2009[22]	2/73	21/165
Wang, S. Y 2011[24]	16/210	22/286

OS= Overall survival

## Discussion

Globally, breast cancer is the leading cancer diagnosed in women. In 2008, the World Health Organization estimated 1,384,155 new cases globally [25]. While surgical removal of the primary tumor remains the mainstay of treatment, the addition of adjuvant therapies based on risk of recurrence have been found to significantly improve the overall prognosis. Specifically in the post-mastectomy setting, while the benefit of adjuvant radiation has become widely accepted as standard of care for patients with tumors >5cm in size, 4 or more positive lymph nodes, or positive margins, the benefit of PMRT for the T1/T2, N1-3+ subgroup remains controversial. Specifically, there is discordance in the published data on risk of LRR in this subgroup of patients. For example, the Vancouver BC PMRT trial demonstrated the benefits seen in patients with 1-3 positive nodes were similar to that of patients with 4 or more positive nodes [26]. Yet the study by Rangan et al, which included patients without radiotherapy, found only 11% of patients with T1 tumors and 17% in T2 patients developed a LRR [27]. Furthermore, practice guidelines for PMRT have not been consistent. The National Comprehensive Cancer Network (NCCN) between 2008-2010 changed its verbiage from ‘consider’ to ‘strongly consider’ PMRT in patients with 1-3 positive nodes [11]. However, the consensus from the St. Gallen breast cancer meeting in 2009 reported that routine adjuvant PMRT was not recommend, and PMRT should be considered only for young patients or those with other poor prognostic factors [28]. We believed that the result of randomized control trial SUPREMO could offer helpful reference to the use and value of PMRT[9]. The merits of randomized control trials are there is no selection bias, but it may expose patients to risky factors. In contrast, cohort study may present selection bias. Because of the recent evolution in the paradigm that radiation therapy not only affects local-regional outcomes, but can have a modest impact on diminishing distant metastasis and thus ultimately have an impact in improving long term overall survival, the outcome of LRR, in and of itself, should be considered as a relevant endpoint, particularly in patients with anticipated longevity. Furthermore, recurrences after mastectomy have the potential to significantly impact the patient’s quality of life [29].

In this meta-analysis assessing the benefit of PMRT on LRR for T1/T2, N1-3+ tumors from 10 studies, the combined pooled RRs of LRR for PMRT was 0.348 (95% CI = 0.254-0.477), suggesting a significant impact of PMRT in reducing LRR (p<0.05), without any significant heterogeneity detected between studies (p=0.380; I^2^ = 6.7%). 

Because there have been implications from other studies that tumor size alone may be the only significant factor driving this benefit size. We found that irrespective of the division by tumor size, the risk of LRR with the addition of PMRT resulted in a pooled RR of 0.330 (95% CI = 0.171 to 0.639) for T1/N1-3+, similar to the benefit of PMRT on the entire T1/T2,N1-3+ cohort; In the T2/N1-3+ subset analysis, the pooled RR with the addition of PMRT was 0.226 (95% CI = 0.121 to 0.424), suggesting that the magnitude of benefit from the PMRT to reduce LRR is slightly greater for patients with larger tumor size.

In the analysis assessing the benefit of PMRT on OS for T1/T2, N1-3+ tumors from 6 studies, the combined pooled RRs of LRR for PMRT was 1.051 (95% CI =1.001 to 1.104). Although this analysis showed the pooled RR between PMRT and no-PMRT group on OS was not significant, there is a trend that patients may benefit from PMRT on OS. Due to the detected heterogeneity, more clinical studies are necessary to clarify if PMRT can improve OS.

Based on the EBCTCG meta-analysis [31], the risk of LRR in patients treated with mastectomy and systemic therapy are lower compared with patients treated with mastectomy only, and systemic therapy can prolongs the survival of patients. In our study, all but one trial included most patients treated with systemic therapy. However, only 2 trials have investigated the role of systemic therapy [32,33]. Duraker et al reported the relative risk between subgroups treated with or without chemotherapy was 1.24 (0.39–3.96), and 1.59 (0.76–3.36) to hormonal therapy [32]. In the study of Wu et al, only hormonal therapy was investigated, and it can significant reduces the risk of LRR in both PMRT and no- PMRT group (p<0.001) [33].

Based on Overgaard subgroup trials [2], the risk of LRR can be reduced significantly with the irradiation to regional nodes, but with increased toxicity. In the MacDonald trial, the addition of a SCV field increased the amount of lung, normal lymphatics, vasculature, and bone receiving RT, and symptomatic pneumonitis has been seen in <1% of patients treated with tangent RT, but increases to<4.1% with treatment of the regional lymph nodes [22]. Because radiotherapy is associated with potential long-term side effects that may ultimately negatively impact on an individual patient’s quality of life [34,35], it is important to identify patients with a relatively high risk of LRR for PMRT. Studies attempting to identify clinical-pathologic features and risk of LRR have been conflicting. For example, with regards to age as a prognostic factor, Bertheau et al reported that younger patients have a better prognosis [36], Rapitiet al reported that age is not an independent prognostic factor [37] and Tai et al suggest that the association between age and recurrence resembles a U-shaped curve, with the highest LRR occurring at younger and older ages and the lowest risk of LRR occurring at approximately 50 years of age [38]. Similarly, Katz et al found that tumor size of 4 cm or larger; ECE beyond 2mm, and dissection of fewer than 10 nodes increased the risk of LRR in stage T1/T2,N1-3+ patients treated without PMRT [39]. In similar populations of patients, Truong et al reported that a >25% nodal ratio, medially located tumors, and ER-negative hormone receptor status predicted for increased risk of LRR [10], Yang et al found ER-negative tumors to be of greatest predictive value in their cohort [12], and Cheng et al suggest that only tumor size is significant for LRR [30]. Lastly, Wu et al defined T2 tumors, 2 or 3 positive lymph nodes, and hormone receptor negative tumors as risk factors for LRR and recommended PMRT for patient with 2 or more of these adverse factors [11]. Based on different variables assessed in previous studies, a multitude of risk factors for LRR have been postulated such as tumor size, types of treatment delivered, pathologic grade, total number of dissected nodes, number of involved lymph nodes, nodal ratio, lymph node extracapsular extension, location of the primary tumor within the breast and the receptor status for ER, PR, and HER-2. More recent analyses of molecular and genetic markers are also under active investigation to predict LRR [40,41]. At this time, the data do not consistently support a higher risk LRR risk in any narrow subgroups outside of the classic >5cm, >4 nodes, and positive margins to identify patients for PMRT. Thus, additional studies are needed to further define potential risk factors to define subgroups that would most benefit from PMRT.

Due to the insufficient reporting of the various risk factors and tumor characteristics across studies, pooled analysis to assess associations between other clinical-pathologic features (outside of tumor size) and risk of LRR could not be conducted in this study. For our subset analysis by tumor size, our findings suggest that a significant benefit with PMRT for reducing LRR exists for both for T1 and T2 tumors, though the magnitude of this benefit is slightly greater for T2 tumors.

### Limitations of This Study

Our study has some limitations. Firstly, we failed to investigate more factors that may influence LRR for the lack of more detail data. We can only analysis the factor tumor size that may influence the prognosis after PMRT, other factors like age, ER status, PR status cannot be analysis based on trials involved in our study. More clinical trials are needed to elucidate risk factors for LRR to give further guidance on PMRT to patients with T1-T2 and 1-3 positive lymph nodes. Secondly, the studies involved in our analysis are non-randomized cohort studies; through there is no publication bias as we have illustrated above, other bias like selection bias should also take into account. To minimize the influence of selection bias on our analysis, we have referred to Newcastle-Ottawa Scale (NOS) standard to evaluate the quality of each study, only studies with good quality are involved.

## Conclusion

In summary, this meta-analysis of T1/T2, N1-3+ patients demonstrates a significant reduction in LRR with the use of PMRT in patients with 1-3 positive lymph nodes irrespective of primary tumors size. Based on these findings, patients with 1-3 positive nodes with tumors less than 5 cm in size should be considered for PMRT, with the goal of significantly reducing the relatively high risk of LRR. Recommendations for PMRT for T1/T2, N1-3+ patient, should be made in the context of other considerations such as anticipated longevity, patient preferences, and risk versus benefits for the individual patient. Additional prospective data are needed to elucidate risk factors for LRR in the post-mastectomy setting so that patients with these factors can experience the greatest benefit from PMRT.

## Supporting Information

Checklist S1 **PRISMA Checklist.**
(DOC)
